# Serum Selenium Level Predicts 10-Year Survival after Breast Cancer

**DOI:** 10.3390/nu13030953

**Published:** 2021-03-16

**Authors:** Marek Szwiec, Wojciech Marciniak, Róża Derkacz, Tomasz Huzarski, Jacek Gronwald, Cezary Cybulski, Tadeusz Dębniak, Anna Jakubowska, Marcin Lener, Michał Falco, Józef Kładny, Piotr Baszuk, Jerzy Duszyński, Joanne Kotsopoulos, Steven A. Narod, Jan Lubiński

**Affiliations:** 1 Department of Surgery and Oncology, University of Zielona Góra, Zyty 28, 65-046 Zielona Góra, Poland; szwiec72@gmail.com; 2 Read-Gene S.A., 72-003 Grzepnica, Poland; wojciech.marciniak@read-gene.com (W.M.); roza.derkacz@read-gene.com (R.D.); 3 Department of Genetics and Pathology, International Hereditary Cancer Center, Pomeranian Medical University, 71-252 Szczecin, Poland; huzarski@pum.edu.pl (T.H.); jgron@pum.edu.pl (J.G.); cezarycy@pum.edu.pl (C.C.); debniak@pum.edu.pl (T.D.); aniaj@pum.edu.pl (A.J.); marcinlener@poczta.onet.pl (M.L.); baszukpiotr@gmail.com (P.B.); 4 Independent Laboratory of Molecular Biology and Genetic Diagnostics, Pomeranian Medical University in Szczecin, 71-252 Szczecin, Poland; 5 Regional Oncology Centre, 71-730 Szczecin, Poland; falco.miw@op.pl; 6 Department of General and Oncological Surgery, Pomeranian Medical University, 71-252 Szczecin, Poland; jkladny@onet.pl; 7 Nencki Institute of Experimental Biology, Polish Academy of Sciences, 3 Pasteur Street, 02-093 Warsaw, Poland; j.duszynski@nencki.gov.pl; 8 Women’s College Research Institute, Women’s College Hospital, University of Toronto, Toronto, ON M5G 1N8, Canada; Joanne.Kotsopoulos@wchospital.ca (J.K.); steven.narod@wchospital.ca (S.A.N.); 9 Dalla Lana School of Public Health, University of Toronto, Toronto, ON M5T 3M7, Canada

**Keywords:** selenium, breast cancer, mortality

## Abstract

In a recent prospective study, we reported an association between a low serum selenium level and five-year survival among breast cancer patients. We now have updated the cohort to include 10-year survival rates. A blood sample was obtained from 538 women diagnosed with first primary invasive breast cancer between 2008 and 2015 in the region of Szczecin, Poland. Blood was collected before initiation of treatment. Serum selenium levels were quantified by mass spectroscopy. Each patient was assigned to one of four quartiles based on the distribution of serum selenium levels in the whole cohort. Patients were followed from diagnosis until death or last known alive (mean follow-up 7.9 years). The 10-year actuarial cumulative survival was 65.1% for women in the lowest quartile of serum selenium, compared to 86.7% for women in the highest quartile (*p* < 0.001 for difference). Further studies are needed to confirm the protective effect of selenium on breast cancer survival. If confirmed this may lead to an investigation of selenium supplementation on survival of breast cancer patients.

## 1. Introduction

Selenium is an essential component of several major metabolic pathways, including the antioxidant defense system and the immune system and selenium is incorporated into 30 different selenoproteins [1,2,3]. Selenoproteins play important roles in anti-oxidation and in DNA stability and may mediate the anti-cancer effect of selenium [4]. Selenium has an effect on cell proliferation and apoptotic cell death in healthy and malignant cells [5]. Low selenium levels have been associated with a high incidence of several different cancer types [3,6] as well as cancer mortality [7]. Selenium intake varies between countries [2,8,9]. The level of selenium is higher in the United States and Canada than in Europe [8]. Randomized controlled trials did not provide clear evidence for an impact of selenium supplementation on cancer incidence or mortality [7], however the majority of randomized trials have been conducted countries with high dietary intakes of selenium (such as Canada and the United States) where selenium deficiency is uncommon [10,11,12,13,14]. 

We have previously reported that in Poland, low serum selenium levels are associated with increased risks of laryngeal, lung and colorectal cancers [15,16]. Information is emerging on the influence of selenium on the prognosis of patients with cancer. A recent Swedish study showed a superior breast cancer-specific survival in patients with a serum selenium in the highest quartile (>100.0 μg/L) compared to the lowest quartile (<81.0 μg/L) (HR 0.60; 95% CI 0.37–0.98) [17]. Sweden, like Poland, is a country with low soil selenium [2,8,9,18]. In Poland, the mean serum selenium level among women is approximately 80–90 μg/L, compared to >130 μg/L in the United States [15,19]. We have reported a relationship between low serum selenium and the five year survival of patients with breast, lung and laryngeal cancer in Poland [20,21,22]. The objective of the current analysis is to report on the 10-year survival of experience of the breast cancer patients in our earlier cohort.

## 2. Materials and Methods

### 2.1. Study Population

Out study included 538 breast cancer patients who were diagnosed between 2008 and 2015 and who were treated at one of two hospitals associated with the Pomeranian Medical University in Szczecin, Poland. The diagnosis of invasive breast cancer was confirmed by biopsy review at a central pathology laboratory in Szczecin. We excluded patients with a past history of breast cancer or another cancer, women with stage IV cancer (metastatic disease at diagnosis) or with pure DCIS. Clinical data were obtained from the review of medical records. Blood samples were collected and extracted DNA was assessed for three founder mutations in *BRCA1* (c.5263_5264insC; c.4035delA; c.181T>G) according to standard protocols All subjects provided written consent for an additional blood sample to be drawn and stored for research purposes. The study was approved by the institutional ethics review boards of the host institutions.

### 2.2. Ethical Approval and Informed Consent

The study was conducted in accordance with the Declaration of Helsinki, and was approved by the Ethics Committee of the Pomeranian Medical University in Szczecin—IRB BN-001/174/05.

### 2.3. Analytical Procedures

A blood sample was collected from each participating patient during an outpatient clinic visit. Patients were asked to fast for at least four hours prior to giving blood. Tubes were incubated at room temperature for minimum 30 min to facilitate clotting and then were centrifuged for 12 min. Serum was aliquoted into new cryovials and deep-frozen at −80 °C. Patients were included in this study if the blood sample was taken within three months of the date of diagnosis and before initiation of treatment. 

Serum selenium levels were measured using a NexION 350D inductively coupled plasma mass spectrometer (Perkin Elmer, Shelton, CT USA). The spectrometer was equipped with Universal Cell Technology (UCT). Selenium isotope ^78^Se was selected for determination by ICP-MS. KED mode with helium (Kinetic Energy Discrimination or KED) was used for reduction of polyatomic interferences. Calibration standards were prepared from 10 µg/mL Multi-Element Calibration Standard 3 (Perkin Elmer) by diluting with blank reagent to the final concentration of 30, 60, 100 and 150 µg/L. Correlation coefficients for calibration curves were always greater than 0.999. Analysis protocol assumed 30-fold dilution of serum in blank reagent. Blank reagent consisted of high purity water (>18 MΩ), TMAH (AlfaAesar, Kandel, Germany), Triton X-100 (PerkinElmer, Shelton, CT, USA), *n*-butanol (Merck, Darmstadt, Germany and disodium EDTA (Sigma Aldrich, Steinheim, Germany). Rhodium was set as internal standard. ClinChek^®^ Serum Control Level I (Recipe, Munich, Germany) was used as a reference material.

Differences in serum selenium levels between the current measurement and the measurement used in our earlier study (17) are due to changing the internal standard-replacement of germanium by rhodium and by introduction of matrix-matched external calibration.

### 2.4. Statistical Analysis

The mean value of serum selenium was estimated for various subgroups and differences in selenium levels were assessed for statistical significance using the Student’s *t* test and one-way ANOVA. Patients were followed from the date of diagnosis until the first of death from breast cancer, death from another cause or the date last known alive. Actuarial survival rates were estimated by the Kaplan–Meier method and differences in survival were compared using the log-rank test. We estimated hazard ratios (univariable and multivariable) for breast cancer-specific survival and for all-cause mortality using Cox-regression analysis. The multivariable model included all variables that were significant predictors of death in the univariable model (*p* < 0.1). In the multivariable model, a *p*-value of <0.05 was considered to be statistically significant. The analysis was conducted using TIBCO Software Inc. (2017) (Palo Alto, CA USA) and Statistica (data analysis software system), version 13 (StatSoft, Krakow, Poland; http://statistica.io; accessed on 10 November 2020).

## 3. Results

### 3.1. General Characteristics of the Study Population

There were 538 breast cancer patients included in this study (Table 1). The median age of diagnosis was 62 years (range 26–89 years). A germline *BRCA1* mutations was present in 11.5% of the patients. The majority of cases were estrogen receptor (ER) positive (69.1%); 60.8% had negative lymph nodes and 92% had a tumor of size less than 5 cm. 52.4% of the patients received chemotherapy, 57.3% received radiotherapy and 67.5% received tamoxifen.

### 3.2. Serum Selenium Level—Subgroup Analysis 

The mean selenium level was 86.2 μg/L (range 52.12–171.55 μg/L). The mean selenium levels increased with age (*p* < 0.0001), but did not vary according to smoking history or tumor factors (tumor size or nodal status) (*p* > 0.05). The mean serum selenium level in subgroups are presented in Table 1.

### 3.3. All-Cause Mortality, Breast Cancer-Specific Mortality 

After a mean follow-up period of 7.9 years, 121 of the 538 patients had died (22.5%); 81 deaths (66.9%) were from breast cancer, 10 deaths (8.7%) were from other cancers and 25 deaths (20.7%) were from other causes. For five patients (4.1%), the cause of death was unknown. 

The overall 10-year survival rate was 76.2% for the entire cohort. The 10-year overall survival rate was 65.1 % for women with low selenium (quartile 1), was 75.1% for women in quartile 2, was 77.7% for women in quartile 3 and was 86.7% for women in quartile 4 (*p*-long rank < 0.001) (Table 2). 

**Figure 1:**
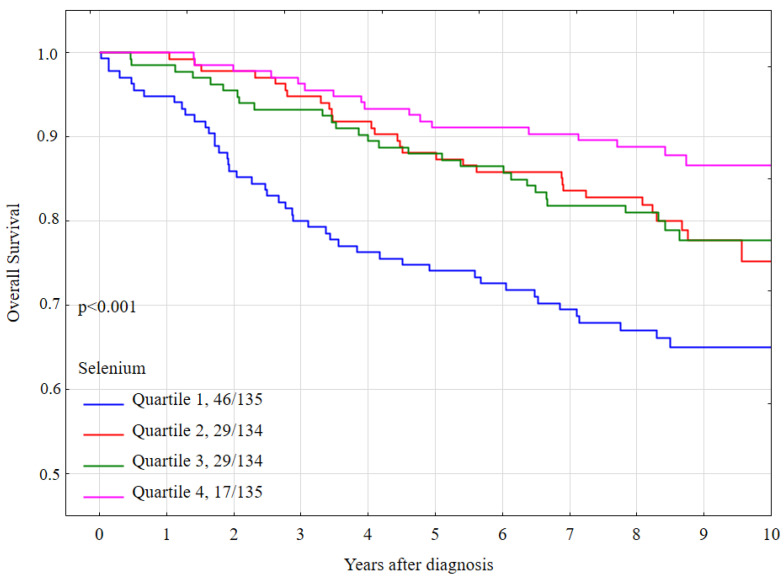
Ten-year all-cause mortality by quartile of serum selenium levels, all women.

Compared to women in the highest quartile (quartile 4) the multivariate hazard ratios (HR) for all-cause mortality were 2.35 (95% CI 1.21–4.55, *p* = 0.01) for quartile 1, 1.52 (95% CI 0.76–3.02, *p* = 0.23) for quartile 2, and 1.95 (95% CI 1.01–3.76, *p* = 0.047 for quartile 3 (Table 4). Overall survival by quartile is presented in Figure 1.

**Figure 2:**
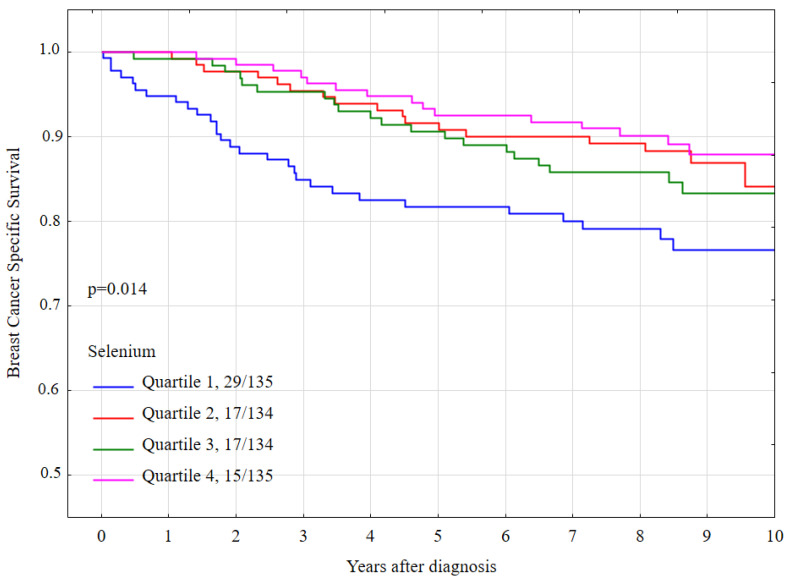
Ten-year breast cancer-specific survival by quartile of serum selenium levels, all women.

The 10-year breast cancer specific survival rates were lower for women with a selenium level in quartile 1 (76.7%) than for women in the other three quartiles (84.2% for quartile 2, 83.4% for quartile 3, 87.9 for quartile 4) and the difference was statistically significant (*p*-long rank = 0.014) (Table 2). Breast cancer-specific survival by quartile of serum selenium is presented graphically in Figure 2.

Compared to women in quartile 4, the univariate hazard ratio (HR) for breast cancer-specific mortality for women in quartile 1 was 2.31 (95% CI 1.24–4.31, *p* = 0.008). (Table 3). Compared to women in quartile 4, the multivariate hazard ratio (HR) for breast cancer-specific mortality for women in quartile was 1.56 (95% CI 0.72–3.40) and this difference did not reach statistical significance (*p* = 0.26) (Table 4). 

## 4. Discussion

In the present study of 538 breast cancer patients from Szczecin (Poland) we confirmed that a low serum selenium level (i.e., below 76.8 μg/L) at the time of a breast cancer diagnosis was associated with increased risk of death in the 10 years following diagnosis. The 10-year survival rate was 57.1% for women with a selenium level in the lowest quartile, compared to 86.7% for women in the highest quartile. The data confirms that in our previous study of shorter term survival in the same group of patients [20]. Sandsveden et al., published similar results, they included 1066 breast cancer cases [17]. Those authors also observed a significant difference in overall survival (HR 0.63; 95% CI 0.44–0.89) and in breast cancer-specific survival (HR 0.60; 95% CI 0.37–0.98) for patient in the the highest serum selenium quartile (>100.0 μg/L) compared to the in the lowest quartile (<81.0 μg/L). The mean value of selenium level was similar in the Polish cohort (86.2 μg/L) and in the Swedish cohort (92.2 μg/L) [17].

Three publications have presented the association between dietary selenium (rather than circulating selenium levels) and survival in women with breast cancer [23,24,25]. A second study in Sweden showed a positive correlation between high levels of selenium in the diet and improved survival in patients with breast cancer [23]. However, two studies in the U.S. did not confirm this association [24,25]. 

Due to the geographic variability in soil selenium levels, dietary intake in Poland and Sweden tends to be lower than in the United States (US) and this may be the reason why study results differ. Data from the Nutritional Prevention of Cancer (NPC) trial suggest that the protective influence of selenium may be limited to individuals with reduced selenium levels [10]. In our study mean selenium level was 86.2 μg/L, compared to the United States where the mean serum selenium for women aged 40 or older is 134.7 μg/L [19]. 

It is not well understood how selenium levels affect breast cancer prognosis. It is believed that selenium incorporation into selenoproteins (in the form of selenocysteine) prevents from oxidative damage and reduces cancer risk. There are also several other functions of selenoproteins that may impact upon prognosis including a role in immunity and inflammation [3]. Many in vivo and in vitro reports have presented that selenium may perhaps avert cancer through affecting cell proliferation, apoptosis, oxidative stress and immunity (reviewed in [4,26]). 

All study participants were fasting before blood sample collection for selenium level assessment. The measurement was conducted prior to treatment. Also, none of the host factors (e.g., nodal status) or treatments received (e.g., chemotherapy) were associated with selenium levels it is likely that the association is due to unrecognized confounding. 

Our study has several limitations. We had no data on BMI status. Selenium was measured only once and a single serum measurements reflects short-term selenium intake. Although the patient cohort was relatively large the small sample sizes for various subgroups were relatively small and we were not well-powered in our subgroup analyses. We saw a significant association between selenium and breast cancer survival only in the univariate analysis. The association was restricted to women with a low selenium level and a trend in survival across the four quartiles was not observed.

## 5. Conclusions

In summary, in this extension of our previous study, we confirm that a low selenium level might contribute to worse survival and for women with breast cancer. Future studies in other geographic regions with low soil selenium levels should be done to confirm our findings. If confirmed, a study could be conducted to evaluate the impact of selenium supplementation on survival of breast cancer patients.

## Figures and Tables

**Table 1:** Mean serum selenium levels by various treatment, clinical characteristics.

Risk Factor		*n*	%	Mean Selenium Level	*p* ^a^
All		538	100	86.2	
Age					
	26–50	132	24.5	85.1	<0.001 ^b^
	51–60	186	34.6	88.4	
	61–70	146	27.1	87.6	
	≥71	74	13.8	80.0	
*BRCA1* mutation					
	Yes	62	11.5	86.5	0.86
	No	476	88.5	86.2	
Lymph node status					
	Positive	193	35.9	86.1	0.77
	Negative	327	60.8	86.4	
	Missing	18	3.3	84.0	
ER status					
	Positive	372	69.1	85.6	0.06
	Negative	148	27.5	88.3	
	Missing	18	3.4	81.9	
Tumor size [cm]					
	0–1.9	307	57.1	87.2	0.13
	2.0–4.9	188	34.9	86.1	
	≥5.0	12	2.2	79.0	
	Missing	31	5.8	80.0	
Radiotherapy					
	Yes	308	57.3	87.0	0.26
	No	190	35.3	85.5	
	Missing	40	7.4	83.7	
Chemotherapy					
	Yes	282	52.4	86.4	0.80
	No	227	42.2	86.1	
	Missing	29	5.4	84.6	
Type of surgery					
	Lumpectomy	162	30.1	88.0	0.12
	Mastectomy	352	65.4	85.9	
	Missing	24	4.5	78.6	
Tamoxifen					
	Yes	363	67.5	86.0	0.42
	No	157	29.2	87.1	
	Missing	18	3.3	81.9	
Vital status					
	Alive	417	77.5	87.5	
	Dead	121	22.5	81.8	<0.001
	Dead of breast cancer	81	66.9	83.6	0.03
	Dead of other cancers	10	8.3	71.0	<0.001
	Dead of any cancers	91	74.4	82.2	0.002
Smoking					
	Yes, current	115	21.4	86.7	0.59
	Yes, past	139	25.8	86.8	
	Never	271	50.4	85.5	
	Missing	13	2.4	90.5	

^a^*p* values were calculated using *t*-Student and One-way ANOVA; ^b^ 51–60 vs. ≥71 years (*p* = 0.002); 61–70 vs. ≥71 years (*p* = 0.006); ± standard deviation; missing data were excluded from the analysis.

**Table 2:** 10-year overall and breast cancer specific survival.

Selenium Quartile *	Overall Survival (OS)	Breast Cancer Specific Survival		
	10-Year (%)	Log-Rank Test	10-Year (%)	Log-Rank Test
All group	76.2	*p*	83.1	*p*
Quartile 1	65.1	<0.001 ^a^	76.7	0.014 ^b^
Quartile 2	75.1		84.2	
Quartile 3	77.7		83.4	
Quartile 4	86.7		87.9	

^a^ Selenium level 1 vs. 4 (*p* < 0.001); 1 vs. 3 (*p* = 0.01); 1 vs. 2 (*p* = 0.01); ^b^ selenium level 1 vs. 4 (*p* = 0.008); 1 vs. 3 (*p* = 0.11); 1 vs. 2 (*p* = 0.03); * Quartile 1 range 52.1–76.7; Quartile 2 range 76.8–85.1; Quartile 3 range 85.2–94.6; Quartile 4 range 94.7–171.5 µg/L.

**Table 3:** Hazard ratio (HR) and 95% confidence intervals (CI) of all-cause and breast cancer-specific mortality by various treatments and clinical characteristics: univariate analysis.

Risk Factor		All-Cause Mortality			Breast Cancer-Specific Mortality		
		HR (95% CI)		*p*	HR (95% CI)		*p*
Age							
	≤50	1.00	Reference		1.00	Reference	
	51–60	1.23	(0.73–2.08)	0.44	1.17	(0.65–2.09)	0.60
	61–70	1.08	(0.62–1.90)	0.79	0.83	(0.43–1.62)	0.59
	≥71	2.98	(1.73–5.12)	<0.001	1.86	(0.95–3.65)	0.07
*BRCA1* mutation							
	No	1.00	Reference		1.00	Reference	
	Yes	0.84	(0.45–1.57)	0.59	0.89	(0.43–1.85)	0.75
Lymph node status							
	Negative	1.00	Reference		1.00	Reference	
	Positive	2.80	(1.91–4.11)	<0.001	3.44	(2.14–5.52)	<0.001
ER status							
	Negative	1.00	Reference		1.00	Reference	
	Positive	0.93	(0.61–1.40)	0.72	0.64	(0.40–1.02)	0.06
Tumor size [cm]							
	0–1.9	1.00	Reference		1.00	Reference	
	2.0–4.9	2.20	(1.46–3.32)	<0.001	2.42	(1.44–4.05)	<0.001
	≥5.0	6.00	(2.67–3.35)	<0.001	6.91	(2.63–18.1)	<0.001
Radiotherapy							
	No	1.00	Reference		1.00	Reference	
	Yes	0.85	(0.57–1.25)	0.40	1.03	(0.63–1.67)	0.91
Chemotherapy							
	No	1.00	Reference		1.00	Reference	
	Yes	1.46	(0.98–2.17)	0.06	2.37	(1.40–4.00)	0.001
Type of surgery							
	Mastectomy	1.00	Reference		1.00	Reference	
	Lumpectomy	0.42	(0.25–0.70)	<0.001	0.34	(0.17–0.67)	0.002
Tamoxifen							
	No	1.00	Reference		1.00	Reference	
	Yes	0.98	(0.65–1.47)	0.92	0.67	(0.42–1.06)	0.09
Smoking							
	Never	1.00	Reference		1.00	Reference	
	Yes, current	0.96	(0.61–1.52)	0.88	0.76	(0.42–1.39)	0.37
	Yes, past	0.82	(0.52–1.28)	0.37	0.92	(0.55–1.54)	0.74
Selenium quartile *							
	Quartile 4	1.00	Reference		1.00	Reference	
	Quartile 1	3.26	(1.87–5.69)	<0.001	2.31	(1.24–4.31)	0.008
	Quartile 2	1.78	(0.98–3.23)	0.06	1.19	(0.59–2.38)	0.62
	Quartile 3	1.77	(0.97–3.24)	0.06	1.45	(0.74–2.83)	0.28

* Quartile 1 range 52.1–76.7; Quartile 2 range 76.8–85.1; Quartile 3 range 85.2–94.6; Quartile 4 range 94.7–171.5 µg/L.

**Table 4:** Hazard ratio (HR) and 95% confidence intervals (CI) of all-cause and breast cancer-specific mortality by various treatments and clinical characteristics multivariate analysis.

Risk Factor		All-Cause Mortality			Breast Cancer-Specific Mortality		
		HR (95% CI)		*p* ^a^	HR (95% CI)		*p* ^b^
Age							
	≤50	1.00	Reference		1.00	Reference	
	51–60	1.38	(0.74–2.54)	0.31	1.34	(0.65–2.77)	0.42
	61–70	1.58	(0.82–3.01)	0.17	1.51	(0.69–3.36)	0.30
	≥71	2.60	(1.28–5.28)	0.008	2.03	(0.80–5.15)	0.13
Lymph node status							
	Negative	1.00	Reference		1.00	Reference	
	Positive	1.97	(1.23–3.16)	0.005	2.14	(1.16–3.94)	0.01
ER status							
	Negative		-		1.00	Reference	
	Positive				0.86	(0.28–2.65)	0.79
Tumor size [cm]							
	0–1.9	1.00	Reference		1.00	Reference	
	2.0–4.9	1.66	(1.05–2.63)	0.03	2.04	(1.14–3.67)	0.02
	≥5.0	3.71	(1.52–9.07)	0.004	6.00	(2.00–17.97)	0.001
Chemotherapy							
	No	1.00	Reference		1.00	Reference	
	Yes	1.35	(0.79–2.33)	0.27	1.86	(0.86–4.01)	0.11
Type of surgery							
	Mastectomy	1.00	Reference		1.00	Reference	
	Lumpectomy	0.76	(0.43–1.34)	0.34	0.79	(0.37–1.68)	0.55
Tamoxifen							
	No		-		1.00	Reference	
	Yes				0.76	(0.26–2.24)	0.62
Selenium quartile *							
	Quartile 4	1.00	Reference		1.00	Reference	
	Quartile 1	2.35	(1.21–4.55)	0.01	1.56	(0.72–3.40)	0.26
	Quartile 2	1.52	(0.76–3.02)	0.23	0.99	(0.46–2.16)	0.99
	Quartile 3	1.95	(1.01–3.76)	0.047	1.35	(0.63–2.87)	0.43

* Quartile 1 range 52.1–76.7; Quartile 2 range 76.8–85.1; Quartile 3 range 85.2–94.6; Quartile 4 range 94.7–171.5 µg/L; ^a^ Mutually adjusted for variables: age, lymph node status, tumor size, chemotherapy, type of surgery, selenium quartile; ^b^ Mutually adjusted for variables: age, lymph node status, ER status, tumor size, chemotherapy, type of surgery, tamoxifen, selenium quartile.

## Data Availability

The data presented in this study is available from the respective author upon request. The data is not publicly available due to privacy restrictions.

## Abbreviations

BRCA1: breast cancer 1 gene; DCIS: ductal carcinoma in situ; CI: confidence interval; ER: estrogen receptor; HR: hazard ratio; OS: overall survival; Se: selenium.
